# Optogenetic Control of Engrafted Human Induced Pluripotent Stem Cell-Derived Cardiomyocytes in Live Mice: A Proof-of-Concept Study

**DOI:** 10.3390/cells11060951

**Published:** 2022-03-10

**Authors:** Jyotsna Joshi, Bing Xu, Michael Rubart, Yun Chang, Xiaoping Bao, Hari P. Chaliki, Luis R. Scott, Wuqiang Zhu

**Affiliations:** 1 Department of Cardiovascular Diseases, Mayo Clinic Arizona, Scottsdale, AZ 85259, USA; joshi.jyotsna@mayo.edu (J.J.); xu.bing@mayo.edu (B.X.); chaliki.hari@mayo.edu (H.P.C.); scott.luis@mayo.edu (L.R.S.); 2 Department of Cardiology, Northern Jiangsu People’s Hospital, Clinical Medical College, Yangzhou University, Yangzhou 225001, China; 3 Department of Pediatrics, Indiana University School of Medicine, Indianapolis, IN 46202, USA; mrubartv@iu.edu; 4 Department of Chemical Engineering, Purdue University, West Lafayette, IN 47907, USA; chang692@purdue.edu (Y.C.); bao61@purdue.edu (X.B.); 5 Department of Physiology and Biomedical Engineering, Mayo Clinic Arizona, Scottsdale, AZ 85259, USA; 6 Center for Regenerative Medicine, Mayo Clinic Arizona, Scottsdale, AZ 85259, USA

**Keywords:** heart failure, stem cells, cardiomyocytes, cell therapy, optogenetics

## Abstract

Background: Cellular transplantation has emerged as promising approach for treating cardiac diseases. However, a poor engraftment rate limits our understanding on how transplanted cardiomyocytes contribute to cardiac function in the recipient’s heart. Methods: The CRISPR/Cas9 technique was employed for stable and constitutive gene expression in human-induced pluripotent stem-cell-derived cardiomyocytes (hiPSC-CMs). Myocardial infarction was induced in adult immunodeficient mice, followed by intramyocardial injection of hiPSC-CMs expressing either CCND2/channelrhodopsin 2 (hiPSC-CCND2^OE^/ChR2^OE^CMs) or CCND2/luciferase (hiPSC-CCND2^OE^/Luci^OE^CMs). Six months later, hemodynamics and intramural electrocardiogram were recorded upon blue light illuminations in anesthetized, open-chest mice. Results: Blue light resets automaticity of spontaneously beating hiPSC-CCND2^OE^/ChR2^OE^CMs in culture, but not that of hiPSC-CCND2^OE^/Luci^OE^CMs. Response to blue light was also observed in mice carrying large (>10^6^ cells) intracardiac grafts of hiPSC-CCND2^OE^/ChR2^OE^CM but not in mice carrying hiPSC-CCND2^OE^/Luci^OE^CMs. The former exhibited single premature ventricular contractions upon light illumination or ventricular quadrigeminy upon second-long illuminations. At the onset of premature ventricular contractions, maximal systolic ventricular pressure decreased while ventricular volume rose concomitantly. Light-induced changes reversed upon resumption of sinus rhythm. Conclusions: We established an in vivo model for optogenetic-based modulation of the excitability of donor cardiomyocytes in a functional, reversible, and localized manner. This approach holds unique value for studying electromechanical coupling and molecular interactions between donor cardiomyocytes and recipient hearts in live animals.

## 1. Introduction

One of the major objectives of cell-based therapy for myocardial repair is to generate new muscle or mimics that can functionally replace damaged myocardium. Towards this goal, many different cell types have been tested in the preclinical animal models and in patients [1,2,3,4]. Data from animal studies and clinical evidence from phase I and phase II cases support the potential efficacy of cell-based therapy for a variety of cardiovascular diseases [1,4]. Several lines of evidence have indicated that engrafted cells remuscularize injured myocardia in mice [5], rats [6,7,8], guinea pigs [9,10], pigs [11], and nonhuman primates [12,13]. Recently, exosomes derived from mouse embryonic stem cells (ESCs) [14] or human cardiosphere-derived cells [15] have been shown to attenuate left ventricular remodeling and to improve cardiac function in myocardial infarction (MI) animals, which suggested the transplanted cells may function through paracrine mechanisms instead of direct remuscularization [16]. Despite the encouraging news in this field, several gaps exist which impart critical barriers to the development of more efficient therapies to repair injured myocardia: (a) none of these strategies have been demonstrated to be able to continuously remuscularize the injured heart in the long term and ultimately replace the transmural scar; (b) thus far, there is no definite evidence to demonstrate to what extent the remuscularization by transplanted cells contributes to the observed functional improvement.

We have recently employed a new strategy to enhance graft size by inducing the proliferation in transplanted cardiomyocytes [17,18]. Specifically, we established a human-induced pluripotent stem cell (hiPSC) line which carries a transgene encoding for human CCND2 driven by cardiomyocyte-specific α-myosin heavy chain (α-MHC) promoters [17]. CCND2-overexpressing, hiPSC-derived CMs (hiPSC-CCND2^OE^CMs) exhibit increased cell cycle activity and cell proliferation compared with genetically naïve hiPSC-CMs expressing wild-type levels of CCND2 (hiPSC-CCND2^WT^CMs). In a mouse model of MI, the number of engrafted cells was tripled in hearts injected with hiPSC-CCND2^OE^CMs compared to those receiving hiPSC-CCND2^WT^CMs four weeks post-MI and transplantation [17]. Six months after transplantation, engrafted cells occupied more than 50% of the scarred region in hearts injected with hiPSC-CCND2^OE^CMs and exceeded the number of engrafted cells in those receiving hiPSC-CCND2^WT^CMs by approximately eight-fold [18]. These data suggest that transgenic CCND2 overexpression in hiPSC-CM grafts constitutes a viable approach to enhance engraftment and restore function in ischemic heart disease. 

The improvement in cardiac function that was seen in mouse hearts transplanted with hiPSC-CMs may result from a direct contribution of donor cell contraction to the host myocardium, provided that the donor cells were electrically coupled to the latter. This may be tested by an optogenetic approach, such as overexpression of a light-sensitive, ion-conducting rhodopsin channel in donor cells. Channelrhodopsin 2 (ChR2) is a light-gated cation channel derived from green algae *Chlamydomonas reinhardtii* and has been expressed in mammalian neurons to mediate precise firing of action potentials in response to blue light pulses and to unveil the neural circuitry for different biophysiological responses [19,20,21]. The channel is sensitive to blue light (450 ± 25 nm), opens rapidly after absorbing a photon and generates high permeability for monovalent and divalent cations [19]. In this study, we employed a CRISPR/Cas9 genome editing tool to establish a new hiPSC line which carries transgenes expressing CCND2 and light-activatable cation channel rhodopsin (channelrhodopsin 2) in their cardiomyocyte derivatives (hiPSC-CCND2^OE^/ChR2^OE^CMs). Six months after transplantation to post-MI mouse hearts, the hiPSC-CCND2^OE^/ChR2^OE^CM grafts were exposed to light of appropriate wavelengths. Our data showed that light-induced activation of the engrafted hiPSC-CMs resulted in arrhythmia and cardiac functional decline, which recovered instantaneously when the light was turned off. These data demonstrated the feasibility of using optogenetic-based modulation of the excitability of the engrafted cardiomyocytes in live animals in a functional, reversible, and localized manner.

## 2. Materials and Methods

All animal protocols were approved by the Institutional Animal Care and Use Committee (IACUC) of the Mayo Clinic (approval code A00004706-19). All animal surgical procedures and euthanasia were performed based on approved IACUC protocol and in accordance with the National Institutes of Health Guide for the Care and Use of Laboratory Animals. 

### 2.1. Construction of Cell Lines via CRISPR/Cas9 Genome Editing Technique

**Figure 1:**
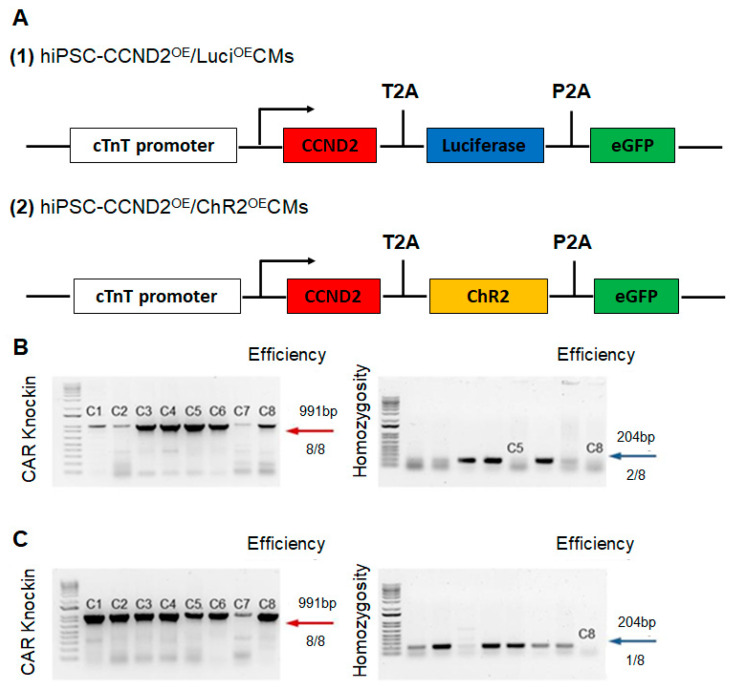
CRISPR-Cas9 based approach for targeted transgene knock-in to the hiPSC line. (**A**) Maps of the cTnT-CCND2-T2A-Luciferase-P2A-eGFP and cTnT-CCND2-T2A-Channelrodpsin 2-P2A-eGFP constructs are illustrated. (**B**) PCR genotyping of *AAVS1* cTnT CCND2 ChR2 eGFP-engineered 19-9-7 hiPSC clones after puromycin selection is shown, and expected PCR product for correctly targeted *AAVS1* site is 991 bp (red arrow) with an efficiency of 8 clones from a total of 8. A homozygosity assay was performed on the knock-in clones, and those without ~240 bp PCR products were homozygous (blue arrow). (**C**) PCR genotyping of *AAVS1* cTnT CCND2 Luciferase eGFP-engineered 19-9-7 hiPSC clones after puromycin selection is shown, and expected PCR product for correctly targeted *AAVS1* site is 991 bp (red arrow) with an efficiency of 8 clones from a total of 8. A homozygosity assay was performed on the knock-in clones, and those without ~240 bp PCR products were homozygous (blue arrow).

Human iPSCs (DF 19-9-7T) were purchased from WiCell. Two new hiPSC lines were established using the CRISPR/Cas9 genome editing technique (Figure 1). The first line carries transgenes expressing CCND2, luciferase, and eGFP driven by cardiomyocyte specific troponin T promoter in their cardiomyocyte derivatives (hiPSC-CCND2^OE^/Luci^OE^CMs). The second line carries transgenes expressing CCND2, light-activatable cation channel rhodopsin (channelrhodopsin 2), and eGFP driven by cardiomyocyte-specific troponin T promoter in their cardiomyocyte derivatives (hiPSC-CCND2^OE^/ChR2^OE^CMs).

An AAVS1 cTnT donor plasmid was built upon the AAVS1-Pur-CAG-eGFR (Addgene, #80945, Watertown, MA, USA), where the CAG promoter was replaced with cTnT promoter. We then cloned CCND2, ChR2 (or Luciferase), and eGFP into the AAVS1 cTnT donor plasmid. The donor plasmid, Cas9 plasmid, and AAVS1 targeted gRNA were co-delivered to iPSCs via nucleofection. To increase cell viability, 10 μM Y27632 was used to treat hiPSCs 3–4 h before nucleofection or overnight. Cells were then singularized by Accutase for 8–10 min, and 1–2.5 × 10^6^ hiPSCs were nucleofected with 3 μg AAVS1 gRNA T2 (Addgene, #41818, Watertown, MA, USA), 4.5 μg pCas9 GFP (Addgene, #44719, Watertown, MA, USA), and 6 μg donor plasmids in 100 μL human stem cell nucleofection solution (Lonza, #VAPH-5012, Bend, OR, USA) using program B-016 in a Nucleofector 2b. The nucleofected cells were seeded into one well of a Matrigel-coated 6-well plate in 3 mL pre-warmed mTeSR plus or mTeSR1 with 10 μM Y27632. After 24 h of incubation, the medium was changed with fresh mTeSR plus or mTeSR1 containing 5 μM Y27632, followed by a daily medium change. When cells were more than 80% confluent, drug selection was performed with 1 μg/mL puromycin for approximately 1 week, and individual clones were picked using a microscope inside a tissue culture hood and expanded for 2–5 days in each well of a 96-well plate pre-coated with Matrigel, followed by PCR genotyping. The genomic DNA of single clone-derived hiPSC was extracted by scraping cells into 40 μL QuickExtract^TM^ DNA Extraction Solution (Epicentre, #QE09050, Middleton, WI, USA). 2×GoTaq Green Master Mix (Promega, #7123, Madison, WI, USA) was used to perform the genomic DNA PCR. For positive genotyping, the following set of primers with an annealing temperature at 65°C was used: CTGTTTCCCCTTCCCAGGCAGGTCC and TCGTCGCGGGTGGCGAGGCGCACCG. For homozygous screening, the following set of primers with an annealing temperature at 60 °C were used: CGGTTAATGTGGCTCTGGTT and GAGAGAGATGGCTCCAGGAA. 

The materials, methods, and complete experimental procedures for nucleofection, construction of donor plasmid, Cas9 plasmid and AAVS1 targeted gRNA have been reported in our previous studies [22,23]. Plasmids pAAV-Syn-ChR2-GFP (Addgene, #58881, Watertown, MA, USA) and Cyclin D2 (Addgene, #8958, Watertown, MA, USA) were purchased from Addgene, while the pCDH EF1alpha Luc2-2A-GFP was kindly gifted from Dr. Joseph Wu, Stanford University. 

### 2.2. Differentiation of Human iPSCs into Cardiomyocytes

hiPSCs were cultured on growth-factor-reduced Matrigel (Corning, #354230, Corning, NY, USA) coated plates and were maintained in mTeSR Plus medium (Stem Cell Technologies, #100-0276, Vancouver, Canada) until they reached 80% confluence. On the first day of differentiation (D0), cells were exposed to 6 µM CHIR99021 (Biogems, #2520691, Westlake Village, CA, USA), a GSK-3 inhibitor, in RPMI 1640 medium (Corning, #10-040-CV, Corning, NY, USA), supplemented with 2% of B27 minus insulin (Gibco, #A18956-01, Billings, MT, USA) (RB minus media), where the media volume in each well of a 6-well plate was 3 mL. On the next day (D1), 2 mL of RB minus media was added while on the third day (D2), 1 mL RB minus media was added on top of the old media. On the fourth day (D3), the old media was pipetted out and cells were rinsed once with sterile PBS, followed by addition of 3 mL of RB minus media supplemented with 3 µM of IWR1, a Wnt inhibitor (Biogems, #1128234, Westlake Village, CA, USA). Then, the culture media was changed to RB minus media alone on Day 5 and 7. On Day 9, media was replaced with RPMI 1640 media, supplemented with 2% B27 (Gibco, #17504-044, Billings, MT, USA) (i.e., RB media). Cardiomyocytes were purified using metabolic selection process, where the regular RB media was pipetted out, cells were rinsed with sterile PBS once and then fed with no glucose RPMI 1640 media (Gibco, #11875-085, Billings, MT, USA), supplemented with 2% B27 supplement and 4 mM of Sodium DL-lactate solution (MilliporeSigma, #L4263, Rockville, MD, USA). After 72 h of metabolic selection, purification media was replaced by the regular cardiomyocyte RB media. These hiPSC-CMs start to beat from day 7 and are cultured for 4 weeks prior to use. 

### 2.3. Assessment of Cell Proliferation and Cell-Cycle Activity in hiPSCs-CMs

After four weeks of the initiation of cardiomyogenic differentiation, hiPSC-CM was stained for the presence of human cardiac Troponin T (hcTnT) and other cell cycle proliferation markers. Nuclei were identified with DAPI staining. Cell proliferation was evaluated via immunofluorescence imaging and labeling of Ki67 (EMD MilliporeSigma, MAB4190, Rockville, MD, USA) expression and quantified as a percentage of the positively stained cardiomyocytes. Cells in S-phase of cell cycle were identified via analyses of Bromodeoxyuridine (BrdU; Abcam, #ab6326, Boston, MA, USA) incorporation and quantified as a percentage of the positively stained cardiomyocytes. Similarly, cells undergoing cytokinesis were identified via Aurora B (Abcam, #ab3609, Boston, MA, USA) expression and quantified as a percentage of the positively stained cardiomyocytes. Likewise, cells in M-phase of cell cycle were identified via analyses of phosphorylated histone H3 (PH3; EMD Millipore Sigma, #06-570, Rockville, MD, USA) expression and quantified as the percentage of positively stained cardiomyocytes.

### 2.4. Assessment of the Beating Activity of hiPSC-CCND2^OE^/ChR2^OE^CMs with Blue Light

The beating activity of hiPSCs-ChR2-CCND2^OE^-CMs was evaluated using bright field microscopy upon blue light illumination (Prizmatix, #UHP-T-460-DI, Holon, Israel), having the centroid of 460 nm and full width of half maximum (FWHM) of 24 nm. Cardiomyocyte beating was analyzed before, during, and after illumination of the blue light irradiated from 4 cm far, with the light intensity of 1.6 mW/mm^2^. The light intensity (power per unit area) was calculated using the following formula:

(1) $$\mathrm{Light}\;\mathrm{intensity}=\frac{\mathrm{Maximum}\;\mathrm{LED}\;\mathrm{Power}\;\left(2W\right)}{\pi *\left({\mathrm{radius}\;\mathrm{of}\;\mathrm{the}\;\mathrm{beam}}^{2}\right)}$$

Maximum light-emitting diode (LED) power was measured using an optical power meter (PM 125D, Thorlabs, Newton, NJ, USA), having the capacity to measure power from 2mW to 10W for 190 nm to 20 µm wavelengths of light.

### 2.5. Calcium and Voltage Mapping of hiPSC-CCND2^OE^/ChR2^OE^CMs

Calcium transients were mapped using Rhod-2AM (Thermo Fisher, #R1245MP, Waltham, MA, USA) dye while action potentials were mapped using RH-237 dye. Rhod-2AM (Thermo Fisher, #S1109, Waltham, MA, USA) dye was reconstituted in DMSO to make 1 mg/mL of stock solutions. The stock solution was diluted in Pluronic acid (Gibco, #24040032, Billings, MT, USA) in a 1:1 ratio. The diluted dye was then added to dye-free Tyrode’s solution to obtain a 10 mM solution. Similarly, RH-237 dye was dissolved in DMSO to make 1 mg/mL stock solutions. The dissolved dye was added to Tyrode’s solution at a concentration of 5 mM. For mapping of calcium transients, cells were washed 3 times with warm Tyrode’s solution followed by incubation with the diluted dye (10 mM) for 15 min at 37 °C. Cells were washed 3 times with Tyrode’s solution and then mapped immediately. For mapping of membrane voltage, cells were washed 3 times with dye-free Tyrode’s solution, followed by incubation with 5 mM of RH237 dye for 5 min at 37 °C. Finally, cells were washed 3 times with Tyrode’s solution and mapped immediately. Calcium or voltage signals were mapped using MiCAM03 N256 Single Camera System (Scimedia Ltd., Costa Mesa, CA, USA) and analyzed using BV Workbench (Version 2.7.2, Scimedia Ltd., Costa Mesa, CA, USA). 

### 2.6. Myocardial Infarction and Cell Transplantation

Experimental mice were housed in the animal facility managed by Mayo Clinic’s Department of Comparative Medicine. Myocardial infarction was surgically induced in 8- to 10-week-old NOD SCID mice (Both male and females, 22–28 g, The Jackson Laboratory, 001303). First, mice were partially anesthetized in isoflurane chamber (2%), then intraperitoneally injected with ketamine and xylazine and then intubated and ventilated with oxygen and 2% isoflurane. Next, left thoracotomy was performed followed by a left anterior descending coronary artery ligation using a 6-0 non-absorbable suture. Four groups were established: (1) mice that did not receive any intramyocardial injection (sham control, n = 8), (2) mice that received intramyocardial injections of 15µL PBS (PBS, n = 6), (3) mice that received 10^6^ hiPSC-CCND2^OE^/Luci^OE^CMs (n = 10), and (4) mice that received 10^6^ hiPSC-CCND2^OE^/ChR2^OE^CMs (n = 10) immediately after coronary artery ligation. Specifically, mice receiving cell injection were injected at 3 regions (5 µL per region) in the left ventricle, one in the infarct zone and two in the border regions. After the treatment, permanent silk surgical sutures were used to close the chest walls and the skin layers. Buprenorphine SR Lab was given subcutaneously (0.4 mg/kg) immediately after surgery and then every 72 h as needed to control the pain, and mice were inspected carefully for 14 days for any signs of pain and trauma.

### 2.7. Assessment of Engraftment Rate

Cell engraftment rate was determined in mice receiving hiPSC-CCND2^OE^/Luci^OE^CMs using bioluminescence assay. Briefly, animals were anesthetized with 2% isoflurane and injected intraperitoneally with D-luciferin (Perkin Elmer, #122799, Boston, MA, USA) at a dose of 375 mg/kg of body weight. Mice were imaged 10 min after injection and then after every 5 min using IVIS-100 In Vivo Imaging System (Perkin Elmer, #124262, Boston, MA, USA). The highest bioluminescence signal for each mouse was considered for data analysis, which was typically observed 10–15 min after injection. Next, a standard curve was generated by plotting a linear regression curve of cell numbers vs. the respective radiance obtained from the bioluminescence assay for each cell density. The standard curve allowed us to determine the engrafted cell number from the bioluminescence signal obtained from the mice. 

In addition, cell engraftment rates were also determined in mice receiving PBS, hiPSC-CCND2^OE^/Luci^OE^CMs, and hiPSC-CCND2^OE^/ChR2^OE^CMs injections using histological assessment of the cryo-sectioned mouse hearts. In short, after sacrificing the mice, hearts were quickly washed in cold PBS (4 °C), kept overnight in 30% sucrose (4 °C), embedded in tissue embedding medium, and preserved in −80 °C freezer. Heart tissue was sliced from epicardial to endocardial regions using Cryostat machine (Leica, #CM1860, Wetzlar, Germany) with slice thickness of 10 µm. Cells that expressed hcTnT were counted in every 10th serial section of the whole heart, and the total was multiplied by 10 to obtain the number of engrafted hiPSC-CMs per heart. The engraftment rate was calculated by dividing the total number of engrafted hiPSC-CMs by the number of injected cells (1 × 10^6^) and expressed as percentage.

### 2.8. Evaluation of Heart Rhythm and Cardiac Functions

The surgical method for hemodynamic studies is based on the established protocols from previous studies [24,25]. First, mice were anesthetized in isoflurane chamber and then intubated and ventilated in 2% isoflurane. Next, an incision was made to locate the right carotid artery through which an ultra-miniature Pressure-Volume (PV) conductance catheter (Millar, #PVR-1035, Houston, TX, USA) was inserted and advanced into the left ventricle. Then, the animal chest was opened to expose the heart, and the catheter position was adjusted if necessary. A USB-TTL pulser device (Pulser Plus, Prizmatix, Holon, Israel) was programmed to deliver specific pulse train to the LED controller. LED was illuminated from 10 ms–10 s, either as a pulse train (10 ms ON, 10 ms OFF, for 10 s) or as continuous pulses (e.g., 4 s, 6 s, 8 s, and 10 s) with LED intensity of 1.6 mW/ mm^2^. After LED illumination, an incision was made to locate the left jugular vein for saline calibration, which was performed by injecting 5–10 µL of hypertonic saline (10% NaCl) into the vein. The bolus injection causes a rise in the ventricular volume under iso-pressure condition, which allowed us to determine the heart wall conductance (i.e., parallel conductance, Gp). At the completion of the study, we collected blood from the mouse and distributed it among heparin-coated cuvettes of known volumes. The PV catheter was carefully removed from the mice and sequentially dipped in the blood-filled cuvettes, which allowed us to generate a linear regression curve that relates conductance values (MilliSiemens) to the volume units (µL). 

Left ventricular pressure and volume were continuously monitored before, during and after LED illumination via the PV catheter connected to the MPVS-Ultra Single Segment Pressure-Volume Unit (AD Instruments, #880-0168SS, Colorado Springs, CO, USA). The MPVS Ultra has an internal frequency oscillator (20 kHz) that generates small RMS sine wave current (20 µA or 100 µA). The PV conductance catheter has a solid-state pressure sensor and 4 electrodes, where the 2 distal conducting electrodes send AC current and the 2 proximal electrodes sense the potential difference (voltage) within the left ventricle during cardiac cycle. The real-time ventricular pressure, volume, and ECG waveforms were acquired using PowerLab 4/26 (AD Instruments, #PL2604/P, Colorado Springs, CO, USA) with the sampling rate of 4 kHz per channel. PV Loop Workflow module in the LabChart (AD Instruments, Colorado Springs, CO, USA) allowed us to perform pressure and conductance calibration in a stepwise manner which converts voltage signals into respective pressure (mmHg) and conductance (mS) signals. The system computes time-varying ventricular volume from the time-varying total conductance (Gx) using Baan’s equation, which is as follows:

(2) $$\mathrm{Ventricular}\;\mathrm{volume}=\frac{1}{\alpha }{\;\rho L}^{2}\;\left(\mathrm{Gx}-\mathrm{Gp}\right)$$

where ρ = blood resistivity; L = distance between the sensing electrodes; α = Bann’s correction factor; Gx = measured total conductance; Gp = Baan’s parallel/ muscle conductance, which is removed by saline calibration. 

### 2.9. Statistical Analysis

Data are presented as mean ± SEM. Statistical analysis was performed via the Student’s *t*-test for comparisons between two groups, and one-way analysis of variance (ANOVA) with Holm−Sidak post-hoc test for comparisons among three or more groups. *p* < 0.05 was considered statistically significant. 

## 3. Results

### 3.1. CCND2 Overexpression Activates Cell Cycle of hiPSC-CMs and Increases Engraftment Rate in Post-MI Mice

**Figure 2:**
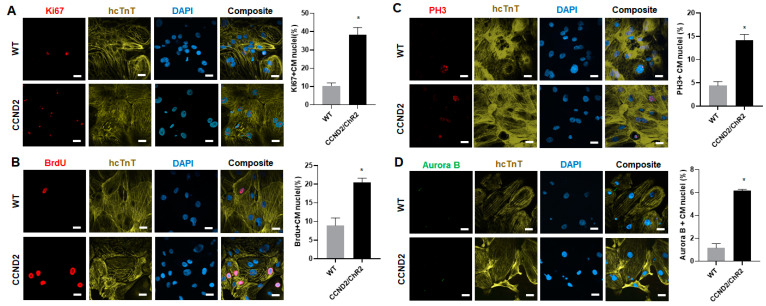
Quantification of cell cycle of CCND2 overexpressing hiPSC-CMs in vitro. The hiPSC-CCND2^WT^CMs (WT) and hiPSC-CCND2^OE^/ChR2^OE^CMs (CCND2) were cultured for 4 weeks after the initiation of cardiomyocyte differentiation, and then immunofluorescently stained for the presence of human cTnT (hcTnT); nuclei were identified via DAPI staining. (**A**) Proliferation was evaluated via immunofluorescence analyses of Ki67 expression in hiPSC-CCND2^WT^CMs and hiPSC-CCND2^OE^/ChR2^OE^CMs and quantified as the percentage of positively stained cells. Scale bar = 20 µm. (**B**) S-phase cells were identified via immunofluorescence analyses of Brdu incorporation in hiPSC-CCND2^WT^CMs and hiPSC-CCND2^OE^/ChR2^OE^CMs and quantified as the percentage of positively stained cells. Scale bar = 20 µm. (**C**) M-phase cells were identified via analyses of PH3 expression and quantified as the percentage of positively stained cells. Scale bar = 20 µm. (**D**) Cells undergoing cytokinesis were identified via Aurora B expression and quantified as the percentage of positively stained cells. Scale bar = 20 µm. *—*p* < 0.05 versus hiPSC-CCND2^WT^CMs by Student’s *t*-test. n = 6 repeats.

We first evaluated the cell cycle in hiPSC-CMs in vitro. The hiPSC-CCND2^WT^CMs (WT) and hiPSC-CCND2^OE^/ChR2^OE^CMs (CCND2) were cultured for 4 weeks after initiation of cardiomyocyte differentiation, and then immunofluorescence staining was performed using antibodies against markers of different cell cycle stages. Our data showed that there was significant upregulation in the expression of Ki67 (cell proliferation marker), Brdu (S-phase marker), PH3 (M-phase marker), and Aurora B (cell cytokinesis marker) in the hiPSC-CCND2^OE^CMs compared to that in hiPSCs-CCND2^WT^CMs (38.21 ± 3.99% vs. 10.43 ± 1.52% for Ki67, *p* < 0.05; 20.40 ± 1.21% vs. 8.90 ± 1.78% for Brdu, *p* < 0.05; 14.17 ± 1.25% vs. 4.40 ± 0.87% for PH3; 6.18 ± 0.09% vs. 1.15 ± 0.34% for Aurora B, *p* < 0.05) (Figure 2). These data suggested that CCND2 expression leads to increased cell cycle in hiPSC-CMs.

**Figure 3:**
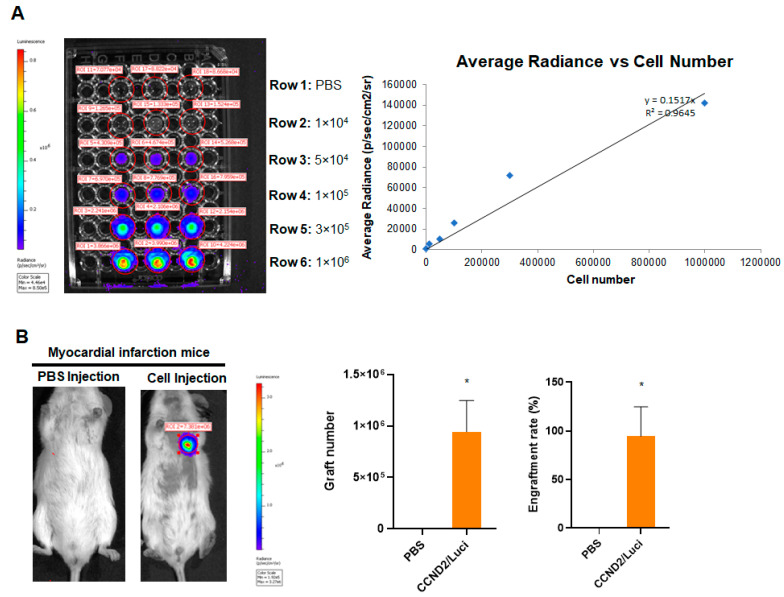
Quantification of graft size in animals receiving CCND2 overexpressing hiPSC-CMs. Immediately after surgically induced myocardial infarction, the mice received intramyocardial injection of 1 million hiPSC-CCND2^OE^/Luci^OE^CMs. Control mice received intramyocardial injection of PBS after myocardial infarction induction. The transplanted cells carried a luciferase reporter plasmid and were of human origin; thus, engraftment was evaluated via bioluminescence imaging at 6 months after cell transplantation. (**A**) A standard curve was generated from bioluminescence measurements of known quantities of hiPSC-CMs. (**B**) Six months after myocardial infarction induction and treatment with intramyocardial injections of PBS (n = 6 animals) or hiPSC-CCND2^OE^/Luci^OE^CMs (n = 10 animals), the mice were injected with luciferin, and bioluminescence images were collected 10 min later. Bioluminescence signal intensity was compared to the standard curve to determine the number of engrafted cells; then, the engraftment rate was calculated by dividing the number of engrafted cells by the total number of cells administered and expressed as a percentage. *—*p* < 0.05 versus PBS-receiving group by Student’s *t*-test.

Next, we evaluated if CCND2-induced cell cycle leads to increased engraftment rate of hiPSC-CMs in post-MI mice. The hiPSC-CCND2^OE^/Luci^OE^CMs carrying a luciferase gene enables in vivo assessment of the number of engrafted cells in live animals at six months after cell transplantation. Hence, we first generated a standard curve by plotting a linear regression curve of cell numbers vs. the respective radiance obtained from the bioluminescence assay for each cell density (Figure 3A). The standard curve allowed us to determine the engrafted cell number from the bioluminescence signal obtained from the mice. Second, we imaged the bioluminescence signal at the chest area of each animal after the intraperitoneal injection of luciferin using the IVIS system. Our data indicated that the average number of engrafted hiPSC- CCND2^OE^/Luci^OE^CMs was ~9.5 × 10^5^, and the average engraftment rate was 94.5% at 6 months after cell transplantation (Figure 3B). It is worthy to note that the engraftment rate in some mice was more than 100%, presumably due to CM proliferation.

### 3.2. Response of hiPSC-CCND2^OE^/ChR2^OE^CMs to Light In Vitro

**Figure 4:**
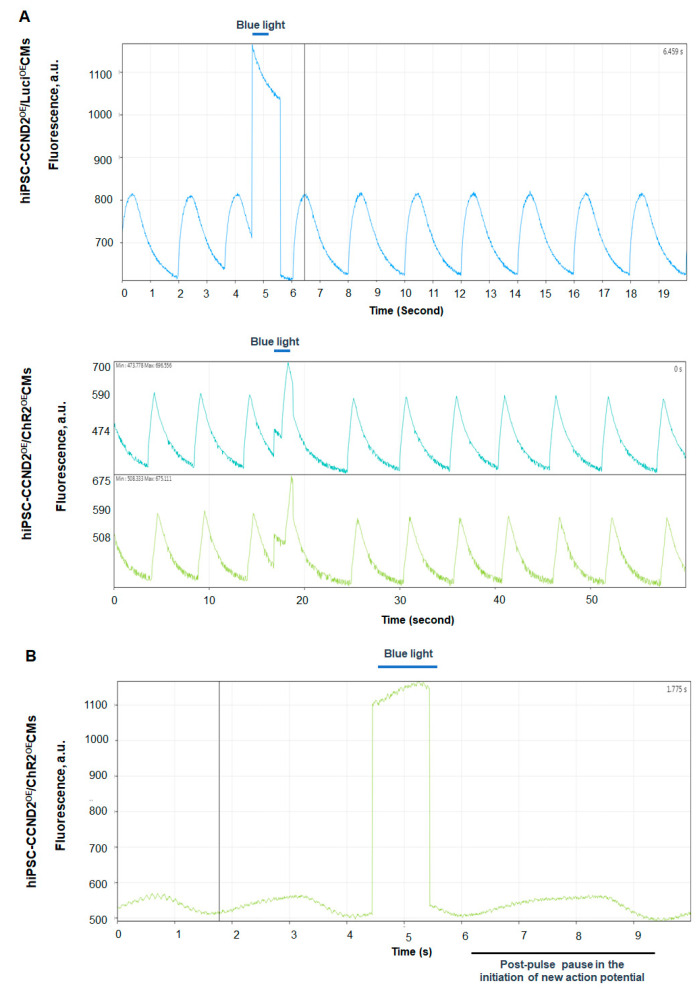
Light-induced changes of calcium transients and action potential of hiPSC-CMs in vitro. (**A**) Calcium mapping. Spontaneous calcium transients in hiPSC-CCND2^OE^/Luci^OE^CMs (upper trace) and hiPSC-CCND2^OE^/ChR2^OE^CMs (middle and bottom traces). Blue light illumination (1 s) did not alter rhythmicity of calcium transients in the hiPSC-CCND2^OE^/Luci^OE^CMs, whereas it delayed the subsequent cycle in hiPSC-CCND2^OE^/ChR2^OE^CMs. (**B**) Voltage mapping. Spontaneous action potentials in hiPSC-CCND2^OE^/ChR2^OE^CMs. Since RH-237 fluorescence decreases with depolarization, the peak and trough fluorescence intensities correspond to the maximal diastolic potential and peak action potential amplitude, respectively. Blue light illumination delayed the onset of the subsequent cycle. Contamination of the fluorescence signals by the blue excitation light prevents their proper interpretation during illumination.

In this study, we utilized genetically engineered hiPSC-CMs that were designed to express blue-light-sensitive ChR2 cation-selective channels. To elicit an action potential in optically excitable cells, such as cardiomyocytes, the optimal number of channel proteins must become activated upon illumination with light of suitable wavelength, and the activated channels must carry sufficient photocurrent to depolarize the cell to the action potential threshold. We evaluated the function of ChR2 in hiPSC-CMs in vitro. Bright field imaging showed that blue light illumination (≥1 s, 1.6 mW/mm^2^) increased the beating rate of hiPSC-CCND2^OE^/ChR2^OE^CMs, while turning off the light caused a delay in resuming their beating automaticity (Video S1). The same illumination protocol did not disrupt rhythmic beating in hiPSC-CCND2^OE^/Luci^OE^CMs (Video S2). To determine if these light-specific responses of hiPSC-CCND2^OE^/ChR2^OE^CMs are due to the changes in the electrophysiology of the hiPSC-CMs, we evaluated the calcium-transient and membrane-potential responses to blue light illumination. Our data from the optical mapping of calcium transients (Figure 4A) and voltage signals (Figure 4B) showed that blue light illumination indeed reset the automaticity of the hiPSC-CCND2^OE^/ChR2^OE^CMs, but not that of hiPSC-CCND2^OE^/Luci^OE^CMs. These data indicated that blue-light-induced alterations in automaticity require the presence of functional ChR2 channels. 

Mattis et al. have reported that activation of ChR2 channels induces around 1 nA of peak inward photocurrent, within <10 ms of the onset of blue light illumination, and maintains a steady state photocurrent of <0.3 nA when illuminated with constant blue light for 1 s [26], indicative of desensitization [19]. Thus, upon continuous illumination, ChR2-expressing hiPSC-CMs will initially induce peak photocurrent to create rapid initial depolarization but will also maintain steady-state photocurrent to create sustained depolarization. Thus, the beating of the ChR2-expressing hiPSC-CMs during blue light illumination is the function of not only of Na^+^, K^+^, and Ca^2+^ ion channels but also of ChR2-light-induced cation channel. Hence, the increased beating rate observed with constant light illumination (≥1 s) in our study is because of ChR2 channels that open and create sustained depolarization in the cells. On the other hand, the observed delay in the resumption of intrinsic beating automaticity of hiPSC-CCND2^OE^/ ChR2^OE^CMs with light off is potentially due to the shift in the electrochemical gradient of the cells due to sustained depolarization, which thereby would have prolonged the repolarization and refractory periods of these cardiomyocytes. 

Taken together, these findings are compatible with the notion that blue-light-induced opening of cation-selective ChR2 channels causes sustained depolarization of cardiomyocyte membranes, sufficient to enhance beating automaticity, which is in agreement with the previous study [27], while the post-light contractile pause reflects functional recovery of the intrinsic pace, making the mechanism upon ChR2 channel deactivation.

### 3.3. In Situ Heart Rhythm and Left Ventricle Hemodynamics in Recipient Mice upon Light Exposure

**Figure 5:**
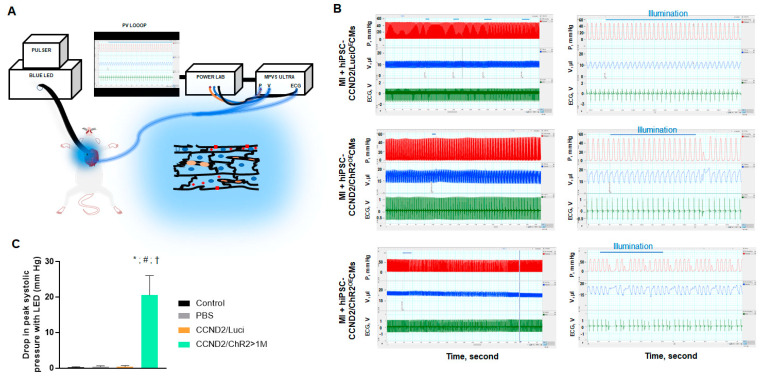
Light-induced changes of heart rhythm and left ventricle hemodynamics in mice. (**A**) Illustration of the optogenetic system. Red squares, ChR2 channels; red plus signs, membrane depolarization; blue ellipsoids, cardiomyocyte nuclei; orange ellipsoids, capillaries. (**B**) Recordings of left ventricular pressure (red), volume (blue), and electrogram (green) in anesthetized, open-chest NOD/SCID mice 6 months after permanent coronary artery ligation and intracardiac injection of hiPSC-CCND2^OE^/Luci^OE^CMs (upper panels) or hiPSC-CCND2^OE^/ChR2^OE^CMs before, during, and after blue light illumination (middle and lower panels). Center panels show tracings from the same animals on the left (boxed region) at expanded time scales. Horizontal blue lines indicate duration of blue light pulses. Premature ventricular complexes (PVC) were recorded with light illumination in mice receiving hiPSC-CCND2^OE^/ChR2^OE^CMs. The arrhythmic effect and decline in cardiac function were mostly transient (row 2, for 3 mice), but lasted for up to 2 min (row 3, 1 mouse). X, Y labels and values are added to improve readability, (Left) LabChart Scale 500:1, (Right) Scale 20:1. (**C**) At the onset of PVC, reduction in systolic ventricular pressure (P_max_) was recorded. *—*p* < 0.05 versus sham control group; #—*p* < 0.05 versus PBS injection group; †—*p* < 0.05 versus CCND2^OE^/Luci^OE^ group. n = 8 mice in sham control; n = 6 mice in PBS injection group; n = 10 mice in hiPSC-CCND2^OE^/Luci^OE^CMs group; n = 10 mice in hiPSC-CCND2^OE^/ChR2^OE^CMs group.

We checked the light-induced response in live animals carrying grafts of hiPSC-CCND2^OE^/ChR2^OE^CMs or hiPSC-CCND2^OE^/Luci^OE^CMs (Figure 5A). Of 34 mice overall, 8 mice were sham control, 6 mice received PBS injection, 10 mice received injections of a total of 10^6^ hiPSC-CCND2^OE^/Luci^OE^CMs, and 10 mice received injections of a total of 10^6^ hiPSC-CCND2^OE^/ChR2^OE^CMs immediately after coronary artery ligation. Of the 10 infarcted mice subjected to intracardiac hiPSC-CCND2^OE^/ChR2^OE^CM injections, only 4 mice exhibited arrhythmias and changes in left ventricular pressure that coincided with epicardial blue light illumination (≥4 s, 1.6 mW/mm^2^). No response was observed in the 8 sham control mice or in the 16 mice injected with PBS or hiPSC-CCND2^OE^/Luci^OE^CMs post-MI (upper panel Figure 5B). These results indicate that the functional effects induced by the blue light are mediated by light-activatable ChR2 channels rather than by unspecific illumination effects (e.g., tissue heating, ROS generation). 

Premature ventricular complexes (PVCs) were identified by the occurrence of widened QRS complexes without preceding P waves in the ECG. The four mice carrying large grafts of hiPSC-CCND2^OE^/ChR2^OE^CMs (e.g., >1 million CMs) exhibited single or repeated monomorphic PVCs during and/or immediately after light illumination (examples are shown in the middle and lower panels of Figure 5B, respectively,). During PVCs, maximal systolic ventricular pressure (P_max_) declined by > 20 mmHg, while ventricular volume increased steadily (Figure 5C). In the subsequent cardiac cycle, systolic pressure rises above the baseline by 5 mmHg, owing to elastic recoil properties of cardiac tissue with increased preload (row 2, Figure 5B). This phenomenon may be explained as heart contraction force is increased with increased preload since the stretched ventricular wall recoil back to their resting state with greater force to push out more blood through the aorta, commonly known as Frank Starling Mechanism. In one mouse carrying a large graft of hiPSC-CCND2^OE^/ChR2^OE^CMs, we observed persistence of PVCs occurring predominately in a 4:1 pattern both during and following a 10 s illumination (lower panel, Figure 5B). It is worthy to note that the observed hemodynamic changes were mostly transient (i.e., one to two cardiac cycles). However, none of the four mice injected with hiPSC-CCND2^OE^/ChR2^OE^CMs exhibited significant changes in left ventricular pressure or volume when their heart beating returned to sinus rhythm during continuous illumination. 

### 3.4. Correlation of Light Response to Graft Size

**Figure 6:**
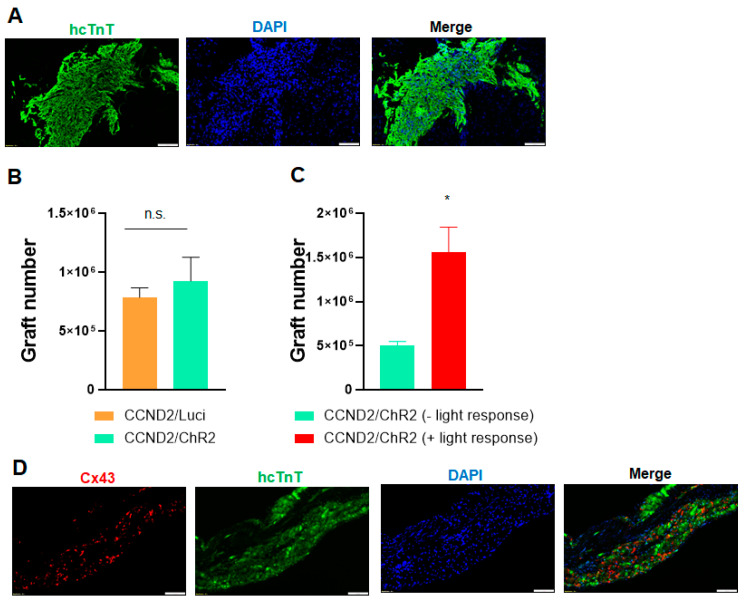
Immunohistological analysis of engrafted hiPSC-CMs. (**A**) Heart sections from light-responsive mice that had been sacrificed 6 months after myocardial infarction induction and treatment with PBS and hiPSC-CMs were stained for the presence of hcTnT. Nuclei were counterstained with DAPI. Scale bar = 100µm. (**B**,**C**) The engraftment rate was calculated by dividing the number of cells that expressed hcTnT by the total number of cells administered and expressed as a percentage. *—*p* < 0.05 versus mice receiving hiPSC-CCND2^OE^/ChR2^OE^CMs but not responsive to light; Student’s *t*-test. n = 10 mice in hiPSC-CCND2^OE^/Luci^OE^CMs group; n = 10 mice in hiPSC-CCND2^OE^/ChR2^OE^CMs group (n = 6 not responsive to light and n = 4 responsive to light). n.s. indicates no significant difference. (**D**) Engrafted hiPSC-CCND2^OE^/ChR2^OE^CMs were identified in mouse hearts at 6 months after myocardial infarction induction and cell transplantation via immunofluorescent staining for the expression of human cTnT. Both transplanted cardiomyocytes and native cardiomyocytes were stained with antibodies against Connexin-43. Images were from heart sections of light-responsive mice. Scale bar = 100 µm.

As discussed above, only four mice receiving hiPSC-CCND2^OE^/ChR2^OE^CM showed arrhythmic and hemodynamic response, while the other six mice receiving the same type and number of cells did not show any response. To understand if the differential response is related to graft size, we performed immunohistological staining to check the engraftment rate of hiPSC-CMs in these animals. Histological studies showed that there is no significant difference for the engraftment rate between the 10 mice receiving hiPSC-CCND2^OE^/Luci^OE^CMs and the 10 mice receiving hiPSC-CCND2^OE^/ChR2^OE^CMs (7.85 × 10^5^ ± 0.74 × 10^5^ vs. 9.25 ×10^5^ ± 2.03 × 10^4^; *p* > 0.05) (Figure 6A,B). We further compared the engraftment rate in the mice receiving hiPSC-CCND2^OE^/ChR2^OE^CMs, and our data indicated the engraftment rate is significantly higher in these four responsive mice compared to the six non-responsive mice (1.56 × 10^6^ ± 2.28 × 10^5^ vs. 5.04 ×10^5^ ± 4.11 × 10^4^; *p* < 0.05) (Figure 6C). These data suggest that a large graft of hiPSC-CMs is necessary to have a significant impact on the cardiac electrophysiology and/or function of recipient mice. 

In mammalian hearts, myocardial contractions are coordinated primarily through the gap-junction proteins Connexin 40 (Cx40), Connexin 43 (Cx43), and Connexin 45 (Cx45), of which Cx43 is by far the most abundant [28]. Cx43 is expressed in both atrial and ventricular myocytes as well as in the Purkinje fibers, and deficiencies in Cx43 expression or in the organization and distribution of gap junctions have been linked to the development of arrhythmias in patients with heart failure and other cardiomyopathies [29,30,31]. Data from computational modeling of electrical activity of engrafted PSC-CMs suggested that poor gap junctional coupling contributed to the engraftment arrhythmias [32]. To understand the pathogenesis of light-induced cardiac arrhythmia, we performed immunohistological staining to check the expression of gap junctional protein connexin 43 in the engrafted hiPSC-CMs. Our data indicated engrafted hiPSC-CCND2^OE^/ChR2^OE^CM express connexin 43 at 6 months after cell transplantation, but the connexin 43 in the engrafted hiPSC-CMs lost the normal orientation compared to mouse myocardium in the remote zone (Figure 6C), which may contribute to the induction of cardia arrythmia upon light activation in the mice carrying large grafts of cardiomyocytes.

## 4. Discussion

Cellular transplantation has emerged as a promising therapeutic approach for treating cardiac diseases [33,34,35]. However, poor donor cell engraftment has been a major roadblock limiting the progress of this field. The low cell engraftment makes it difficult to study the impact of remuscularization by transplanted cells on the electrophysiology and function in host animal hearts. Our recent study demonstrates that genetic induction of proliferation in transplanted cardiomyocytes enhances graft size [17,18,36]. Specifically, we established an hiPSC line which carries a transgene encoding for human CCND2 driven by CM-specific α-myosin heavy chain promoter [17]. CCND2-overexpressing, hiPSC-derived CMs (hiPSC-CCND2^OE^CMs) exhibit increased cell cycle compared with genetically naïve hiPSC-CMs expressing wild-type levels of CCND2 (hiPSC-CCND2^WT^CMs). Intramyocardial injection of hiPSC-CCND2^OE^CMs resulted in significantly increased graft size in post-MI mice and pigs [18,36]. At 6 months after transplantation, the engrafted hiPSC-CCND2^OE^CMs displayed patterns of expression for cTnI, N-Cadherin, Cx43, and Caveolin 3 that were similar to the patterns observed in CMs from the noninfarcted regions of the mouse hearts. The size of grafted hiPSC-CCND2^OE^CMs increased over the 6-month period, and their nuclei density decreased. These data suggest the hiPSC-CCND2^OE^CMs appeared to have matured during this 6-month study [18]. The large cardiomyocyte grafts provide a unique system to study the interaction of donor cells and host heart. In this study, we engineered a new hiPSC line overexpressing both CCND2 and ChR2. CCND2-induced cell cycle leads to the formation of a large graft by transplanted hiPSC-CMs. Upon illumination, ChR2-expressing donor myocytes undergo sustained depolarization but become inexcitable for couple of seconds when light is off. This approach does not ablate grafted cells but rather stops the contraction of the grafted cell, allowing us to assess if donor cells improve the function of the host heart via direct electromechanical coupling. Our data showed that (a) donor cardiomyocytes are electrically coupled to recipient mouse hearts; (b) optogenetic-based manipulation of the donor cardiomyocytes contractility leads to electrophysiological and hemodynamic disorder of recipient heart in a reversible and localized manner; and (c) the impact of light-induced deactivation of grafted cardiomyocytes on recipient hearts is cell-number-dependent. 

Our data indicate that light induces PVC and cardiac functional decline in mice carrying large graft of hiPSC-CCND2^OE^/ChR2^OE^CM. The detailed mechanism underlying these light-induced PVCs is not clear. We have recently performed an optical mapping study on explanted, Langendorff-perfused isolated from the mice carrying the large grafts of hiPSC-CCND2^OE^CMs [18]. The hearts were electrically paced at different cycle lengths. We observed long action potential durations of 80-160 ms over larger infarcted ventricular surfaces of mice receiving hiPSC-CCND2^OE^CM. These long action potential durations were around four times longer than that in non-infarcted regions but were close to APD50 in hiPSC-CM cultures, depicting that long AP in the infarcted regions originated from the engrafted human cardiomyocytes. Interestingly, propagation of those long APs, having a large upstroke and one or more smaller spikes with AP duration of non-infarcted regions, were continuous and followed activation over the non-infarcted regions, showing that engrafted hiPSC-CCND2^OE^CM were electrically coupled to the host mouse cardiomyocytes. Because of the rapid heart rate in mice, the engrafted human cardiomyocytes follow the beating of the mouse cardiomyocytes. However, the human cardiomyocytes retain their beating rhythm during pacing. Based on these findings, we speculate that the origin of light-induced PVCs in mice with intracardiac hiPSC-CCND2^OE^/ChR2^OE^CM grafts may arise from depolarization-induced increase in donor cell automaticity due to ChR2 activation with light, thereby driving the host myocardium. More studies are warranted to confirm this hypothesis.

Apart from the electrical coupling of the graft to the host myocardium, it is also imperative to understand to what extent the engrafted cardiomyocytes contribute to the mechanical functions and impart benefit on the global hemodynamic functions of the host hearts. Although we observed cardiac functional decline in response to light in mice carrying a large graft of hiPSC-CCND2^OE^/ChR2^OE^CM, the functional changes were associated with the onset of PVC, and it normalized when PVC was corrected. The decline of cardiac function in these animals may be caused by multiple reasons, e.g., arrhythmia, the loss of functional myocardium by light-induced inexcitability of engrafted cardiomyocytes, and lack of efficient connection between donor cells or donor/host cell interface. It is difficult to dissect individual contributions in the current study. More electrophysiological and cardiac imaging studies are necessary to precisely understand the mechanism of light-induced cardiac functional decline. However, we found it is difficult to perform cardiac imaging (such as echocardiography) and light exposure on mouse hearts simultaneously owing to the small heart size. Future studies with this optogenetic approach on large animal models are warranted to investigate electrophysiological parameters, action potential propagation, conduction velocity, channel activation time, regional wall motion, and more. Such studies would provide more detailed knowledge on the mechanisms of light-induced cardiac arrhythmia and functional changes on mice carrying the large graft of cardiomyocytes. 

In summary, we developed the first optogenetic-based approach to control the activity of engrafted cardiomyocytes in live animals. This system can be useful to dissect the interaction between engrafted cardiomyocytes and host myocardium, and to determine the impact of engrafted cardiomyocytes on the electrophysiology and global heart function in live animals via selective and reversible modulation of the contractility of these grafted cardiomyocytes.

## Data Availability

The data presented in this study are available on request from the corresponding author.

## Supplementary Materials

The following are available online at https://www.mdpi.com/article/10.3390/cells11060951/s1, Video S1: Illumination of cultured hiPSC-CCND2^OE^/ChR2^OE^CMs with blue light (≥1 s, 460 nm ± 24 nm, 1.6 mW/mm^2^; left panel) triggered repetitive contractions for the duration of the light pulse. Regular, spontaneous contractions resumed within few seconds after light pulse was turned off, Video S2: Illumination of cultured hiPSC-CCND2OE/Luci^OE^CMs with blue light (≥1 s, 460 nm ± 24 nm, 1.6 mW/mm^2^; left panel) had no impact on the cell contraction during and after the light pulse.

## Abbreviations

MI	Myocardial infarction
hiPSC	Human-induced pluripotent stem cells
hiPSC-CM	Human-induced pluripotent stem-cell-derived cardiomyocytes
hiPSC-CCND2^WT^CMs	hiPSC-CMs expressing wild-type levels of CCND2
hiPSC-CCND2^OE^CMs	hiPSC-CMs overexpressing CCND2
hiPSC-CCND2^OE^/ChR2^OE^CMs	hiPSC-CMs overexpressing CCND2 and channelrhodopsin 2
hiPSC-CCND2^OE^/Luci^OE^CMs	hiPSC-CMs overexpressing CCND2 and luciferase
